# The Missing Number

**Published:** 1884-11

**Authors:** 


### THE MISSING NUMBER.

We will pay cash for copies of the INDEPENDENT PRACTITIONER for January, 1884. There must be many in existence whose owners do not particularly desire to preserve them. If they are sent to us with a memorandum of the name and post-office address of the sender, we will gladly pay their full price.

